# TB-Net: A Tailored, Self-Attention Deep Convolutional Neural Network Design for Detection of Tuberculosis Cases From Chest X-Ray Images

**DOI:** 10.3389/frai.2022.827299

**Published:** 2022-04-07

**Authors:** Alexander Wong, James Ren Hou Lee, Hadi Rahmat-Khah, Ali Sabri, Amer Alaref, Haiyue Liu

**Affiliations:** ^1^Vision and Image Processing Research Group, University of Waterloo, Waterloo, ON, Canada; ^2^Waterloo Artificial Intelligence Institute, University of Waterloo, Waterloo, ON, Canada; ^3^DarwinAI Corp, Waterloo, ON, Canada; ^4^Department of Radiology, Niagara Health, McMaster University, Hamilton, ON, Canada; ^5^Department of Diagnostic Radiology, Thunder Bay Regional Health Sciences Centre, Thunder Bay, ON, Canada; ^6^Department of Diagnostic Imaging, Northern Ontario School of Medicine, Sudbury, ON, Canada

**Keywords:** tuberculosis, neural network, self-attention, radiology, efficient, convolutional

## Abstract

Tuberculosis (TB) remains a global health problem, and is the leading cause of death from an infectious disease. A crucial step in the treatment of tuberculosis is screening high risk populations and the early detection of the disease, with chest x-ray (CXR) imaging being the most widely-used imaging modality. As such, there has been significant recent interest in artificial intelligence-based TB screening solutions for use in resource-limited scenarios where there is a lack of trained healthcare workers with expertise in CXR interpretation. Motivated by this pressing need and the recent recommendation by the World Health Organization (WHO) for the use of computer-aided diagnosis of TB in place of a human reader, we introduce TB-Net, a self-attention deep convolutional neural network tailored for TB case screening. We used CXR data from a multi-national patient cohort to train and test our models. A machine-driven design exploration approach leveraging generative synthesis was used to build a highly customized deep neural network architecture with attention condensers. We conducted an explainability-driven performance validation process to validate TB-Net's decision-making behavior. Experiments on CXR data from a multi-national patient cohort showed that the proposed TB-Net is able to achieve accuracy/sensitivity/specificity of 99.86/100.0/99.71%. Radiologist validation was conducted on select cases by two board-certified radiologists with over 10 and 19 years of experience, respectively, and showed consistency between radiologist interpretation and critical factors leveraged by TB-Net for TB case detection for the case where radiologists identified anomalies. The proposed TB-Net not only achieves high tuberculosis case detection performance in terms of sensitivity and specificity, but also leverages clinically relevant critical factors in its decision making process. While not a production-ready solution, we hope that the open-source release of TB-Net as part of the COVID-Net initiative will support researchers, clinicians, and citizen data scientists in advancing this field in the fight against this global public health crisis.

## 1. Introduction

Tuberculosis (TB) remains a devastating global public health crisis, with tremendous on-going negative impact around the world as the leading cause of death for an infectious disease at approximately 1.5 million deaths and 10 million infected each year (World Health Organization, 2020a). Caused by *Mycobacterium tuberculosis* (*M. tb*), a species of pathogenic bacteria, TB is an airborne disease that affects approximately a quarter of the global population, particularly areas of the world faced by poverty and economic distress (World Health Organization, 2020a). More specifically, the most devastating effect of tuberculosis has been on low- and middle-income regions, with two thirds of all tuberculosis infections found in 8 countries (Bangladesh, China, India, Indonesia, Nigeria, Pakistan, Philippines, and South Africa) (World Health Organization, 2020a). Tuberculous is a curable disease, with approximately 85% of infections successfully treated *via* a 6-month drug treatment regimen of different antibiotics.

A very crucial step in the treatment of tuberculosis is screening high risk populations and the early detection of the disease, although tuberculous remains underdiagnosed with an estimated 3 million out of 10 million infected individuals not diagnosed or reported to the World Health Organization (WHO) (World Health Organization, 2020c). Currently, the most widely-used imaging modality used in the screening of tuberculous is chest x-ray (CXR) imaging, which has been shown to be a highly effective and cost-effective screening tool in this scenario (van't Hoog et al., 2012; Li et al., 2018; Diaz et al., 2020). However, one of the biggest limitations with the use of CXR for tuberculosis screening is that it requires experienced human readers such as radiologists or trained clinicians and technicians for interpretation (World Health Organization, 2020a). This problem is exacerbated given the significant shortage of such experienced readers in the most effected countries.

Given this shortage of experienced readers worldwide for CXR interpretation for tuberculosis screening, there has been significant recent interest in artificial intelligence-based TB screening solutions for use in resource-limited scenarios (Chauhan et al., 2014; Hwang et al., 2016; Melendez et al., 2016; Hooda et al., 2017; Lakhani and Sundaram, 2017; Lopes and Valiati, 2017; Vajda et al., 2018; Yadav et al., 2018; Ahsan et al., 2019; Hernandez et al., 2019; Meraj et al., 2019; Nguyen et al., 2019; Pasa et al., 2019; Singh and Hamde, 2019; Rahman et al., 2020). In fact, the most recent WHO guidelines for tuberculosis screening introduced a new recommendation stating that, for those 15 years and older in populations where screening is recommended, computer-aided detection (CAD) may be used in place of human readers for CXR interpretation for tuberculosis screening (World Health Organization, 2020c).

Motivated by this pressing need and the new recommendation by the WHO for the use of CAD for tuberculosis screening, we introduce TB-Net, a self-attention deep convolutional neural network design tailored for TB case screening. More specifically, we leveraged machine-driven design exploration to build a highly customized deep neural network architecture with attention condensers. The TB-Net deep neural network design is not only designed to be high-performing but also highly efficient, which is especially important for practical, operational TB screening given that high-risk regions for TB around the world are those faced by poverty and economic distress, and as such have high resource constraints. In order to validate TB-Net's decision-making behavior, we also conducted an explainability-driven performance validation analysis to show that TB-Net leverages the correct critical factors in making predictions. Furthermore, radiologist validation was conducted with two board-certified radiologists to study the consistency between radiology interpretation and TB-Net's decision-making behavior.

The proposed TB-Net is part of the COVID-Net open source initiative (Gunraj et al., 2020, 2021; Wang et al., 2020; Wong et al., 2020c; Ebadi et al., 2021), which was launched to accelerate advancements in machine learning for tackling different challenges ranging from screening to risk stratification to treatment planning for patients with the severe acute respiratory syndrome coronavirus 2 (SARS-CoV-2). Accordingly to the WHO, it is anticipated that those with both tuberculosis and SARS-CoV-2 infections could potentially experience poorer treatment outcomes, particularly if tuberculosis treatment is interrupted as a result of SARS-CoV-2 infection (World Health Organization, 2020b). Furthermore, tuberculosis and SARS-CoV-2 infection can share similar symptoms (World Health Organization, 2020b). Therefore, effective CAD of both SARS-CoV-2 and tuberculosis infections to support clinicians and front-line healthcare workers can have great potential for improving clinical workflows for tackling these health crises by improving screening, triaging, risk stratification, and treatment planning.

While not a production-ready solution, we hope that the open-source release of TB-Net will support researchers, clinicians, and citizen data scientists in advancing this field in the fight against this global public health crisis.

The article is organized as follows. Section 2 covers recent literature related to tuberculosis detection using deep learning approaches. Section 3 describes the underlying methodology behind the design of the proposed TB-Net, data preparation, explainability-driven performance validation, and radiologist validation. Section 4 presents both the qualitative and quantitative results of the study. Section 5 explores and discusses the efficacy and decision-making behavior of TB-Net both quantitatively and qualitatively, along with radiologist validation. Finally, conclusions are drawn and discussions in broader impact and future directions are presented in Section 6.

## 2. Relevant Work

The shortage of experienced CXR interpreters worldwide for tuberculosis screening, has caused a significant recent interest in the use of artificial intelligence-based TB screening solutions for resource-limited scenarios (Chauhan et al., 2014; Hwang et al., 2016; Melendez et al., 2016; Hooda et al., 2017; Lakhani and Sundaram, 2017; Lopes and Valiati, 2017; Vajda et al., 2018; Yadav et al., 2018; Ahsan et al., 2019; Hernandez et al., 2019; Meraj et al., 2019; Nguyen et al., 2019; Pasa et al., 2019; Singh and Hamde, 2019; Rahman et al., 2020; Rajaraman and Antani, 2020). There are a variety of different approaches, ranging from classical artificial intelligence solutions such as random forests (Melendez et al., 2016) or shape and texture feature vectors (Chauhan et al., 2014; Vajda et al., 2018; Singh and Hamde, 2019), to deep learning approaches using individual CNNs (Hwang et al., 2016; Hooda et al., 2017; Lakhani and Sundaram, 2017; Lopes and Valiati, 2017; Ahsan et al., 2019; Pasa et al., 2019; Rahman et al., 2020) or ensemble CNNs (Hernandez et al., 2019; Rajaraman and Antani, 2020). The advancements of artificial intelligence has even motivated changes to the WHO guidelines for tuberculosis screening, allowing the use of computer-aided detection (CAD) software in place of human readers for CXR interpretation (World Health Organization, 2020c) for those 15 years and older in populations where screening is recommended. CAD software that is able to interpret CXR images is already available (Qin et al., 2021), but suffers from two faults, the first being that the software is usually closed and not available to the general public, and the second being that there is a significant lack of transparency around the software performance and outputs.

Deep learning is a subset of artificial intelligence that has also garnered much attention over the past few decades. The use of deep learning for tuberculosis screening is not a new topic, given the urgency and importance of this task. Most recently, a comprehensive study conducted by Rahman et al. (2020) compared nine different deep convolutional neural network architectures [ResNet-18 (He et al., 2016), ResNet-50, ResNet-101, ChexNet (Rajpurkar et al., 2017), Inception-V3 (Szegedy et al., 2015), VGG-19 (Simonyan and Zisserman, 2015), DenseNet-201 (Huang et al., 2018), SqueezeNet (Iandola et al., 2016), and MobileNet-v2 (Sandler et al., 2019)] for the task of detecting TB patient cases from CXR images, and found the ChexNet deep neural network architecture design (Rajpurkar et al., 2017), a state-of-the-art deep neural network architecture for CXR image analysis, to provide the highest sensitivity and specificity without the use of segmentations. Kim et al. (2020) used a deep CNN in a semi-supervised study, and demonstrated a synergistic effect between the algorithm's classification and radiologist interpretations, further proving the idea that deep learning can be used to perform accurate labeling and classification of CXRs.

However, one issue that tends to arise in deep learning problems is the high resource requirements necessary for the deployment of said systems. Thus, one design consideration behind TB-Net is ensuring that it is high-performing but also highly efficient, which is especially important for practical, operational TB screening given that high-risk regions for TB around the world are those faced by poverty and economic distress, and as such have high resource constraints. In a study by Rajaraman et al., stacked ensemble networks comprised of knowledge-transferred CNNs were leveraged to achieve high performance metrics on multiple CXR datasets (Rajaraman and Antani, 2020), showing that although individual models each performed worse than state-of-the-art architectures, the combination of the top-3 models into a stacked ensemble resulted in above state-of-the-art performance across metrics including accuracy, sensitivity, specificity, and F1 score. However, ensemble methods are computationally expensive and would be hard to deploy in a resource constrained region even given the high potential accuracy it could offer.

Another strategy taken in literature is to try and replicate a radiologist's interpretation procedure when analyzing a screening result. Chandra et al. (2020) proposed a technique leveraging hierarchical feature extraction in which critical features are used in a two-level hierarchy to categorize healthy and unhealthy groups. In the first level, handcrafted geometrical features such as shape, size, and perimeter are used, and in level 2, traditional first order statistical features are used such as texture features, energy, entropy, contrast, and correlation. These features are extracted from segmented lung-fields, where a supervised classification approach is then taken on the extracted features to output a normal or abnormal prediction of a CXR image. However, similar to a CNN ensemble method, the pre-processing steps involved to perform two-level hierarchical decomposition are computationally expensive, and thus would not perform well in a resource constrained environment.

## 3. Methods

In this study, we introduce TB-Net, a self-attention deep convolutional neural network design for detection of TB cases from chest X-ray images. A machine-driven design exploration strategy is leveraged to automatically discover highly customized and unique macro-architecture and micro-architecture designs that make up the proposed TB-Net self-attention deep neural network architecture with attention condensers. Explainability-driven performance validation was conducted to study and validate the decision-making behavior of TB-Net. Finally, radiologist validation was conducted by two board-certified radiologists. The details between data preparation, network design, explainability-driven performance validation, and radiologist validation are described below.

### 3.1. Data Preparation

To train and evaluate the proposed TB-Net, we leveraged the CXR data from a multi-national patient cohort introduced in a study by Rahman et al. (2020), which unified patient cohorts from several initiatives from around the world (Jaeger et al., 2014; Pasa et al., 2019; NIAID, 2020; Rubin et al., 2020). More specifically, the multi-national patient cohort consists of patient cohorts curated by the Department of Health and Human Services in Montgomery County, Maryland, USA, Shenzhen No. 3 People's Hospital in China, the National Institute of Allergy and Infectious Diseases in the USA, as well as the Radiological Society of North America. This multi-national patient cohort represents one of the largest, most diverse patient cohorts for exploring computer-aided tuberculosis screening, to the best of the authors' knowledge.

**Figure 1:**
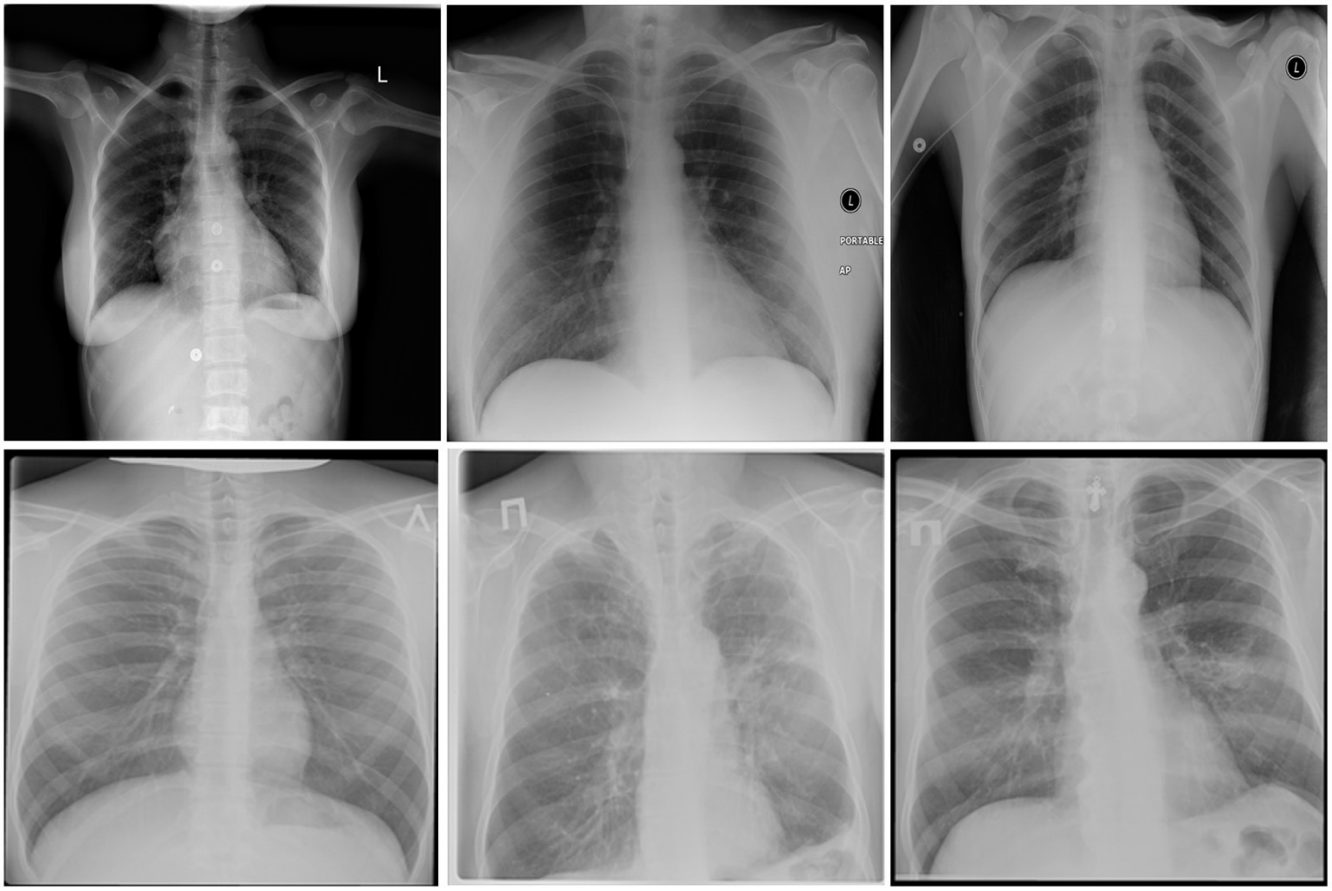
Example chest X-ray images from the multi-national patient cohort introduced by Rahman et al. (2020): **(top)** TB negative patient cases and **(bottom)** TB positive patient cases.

**Figure 2:**
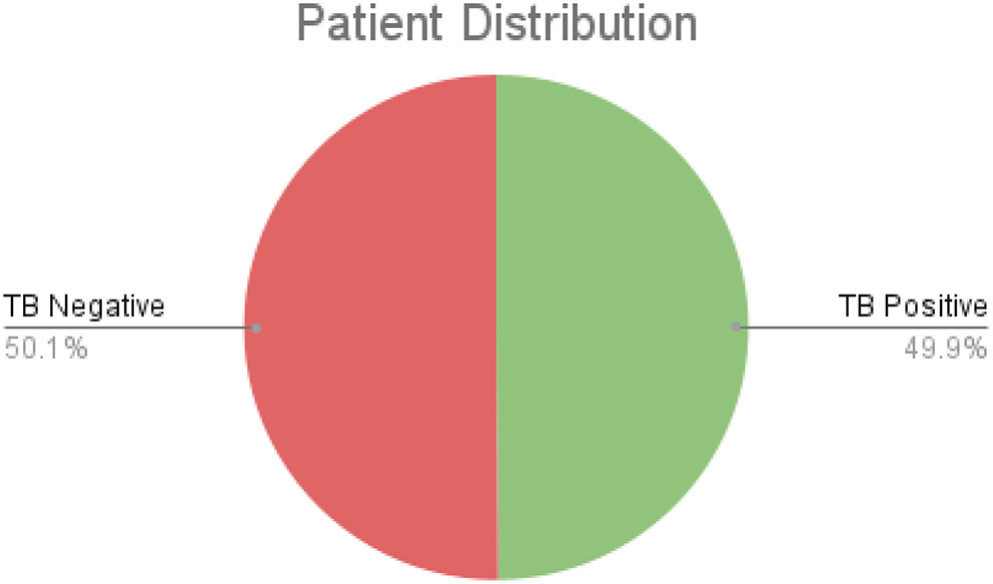
The comparison of TB positive vs. TB negative samples in the dataset. As shown, the split is roughly even.

After additional image quality screening of the CXR images, the CXR data used in this study comprises 6,939 CXR images. In terms of data distribution, there are a total of 3,461 CXR images from TB positive patients and 3,478 CXR images from TB negative patients. The training, validation, and test data consist of 80, 10, and 10% of the patient cases randomly selected from the multi-national patient cohort, respectively. To facilitate for the training and evaluation of TB-Net, the CXR images were resampled to 224 ×224 and mean imputation was performed on the top left-hand and top right-hand corners to mitigate the presence of embedded markings found in the CXR images. Example CXR images from the multi-national patient cohort used in this study for both TB negative and TB positive patient cases are shown in Figure 1. Figure 2 shows the patient distribution between TB positive and TB negative samples in the full dataset.

All data generation and preparation scripts are available in an open source manner at https://github.com/darwinai/TuberculosisNet.

### 3.2. Network Design

The proposed TB-Net self-attention deep neural network architecture design was constructed using a machine-driven design exploration strategy using the aforementioned CXR data from the multi-national patient cohort. More specifically, we leverage the concept of generative synthesis (Wong et al., 2018) to determine the macro-architecture and micro-architecture designs of a deep neural network architecture tailored for the task of TB case detection from CXR images. In generative synthesis, the automatic discovery of the macroarchitecture and microarchitecture designs are posed as a constrained optimization problem, where the goal is to find the optimal generator that generates deep neural network architectures that maximizes a given universal performance function under a set of constraints:

The designs are discovered automatically using an optimal generator G that is capable of, given a set of seeds *S*, generating deep neural network architectures {*N_s_*|*s*∈*S*} that maximize a universal performance function U (e.g., Wong, 2018) under a set of constraints defined by an indicator function 1_*r*_(·),


(1) $$G=\underset{G}{\max}\;U(G(s))\;\;\text{subject}\;\text{to}\;\;1_{r}(G(s))=1,\;\;\forall s\in S.$$

where G denotes the generator, *S* denotes a set of seeds, {*N*_*s*_|*s*∈*S*} denotes a set of deep neural network architectures based on the set of seeds, U denotes a universal performance function U (e.g., Wong et al., 2018), and 1_*r*_(·) denotes an indicator function that defines a set of constraints. When building TB-Net, the set of constraints specified were: (1) sensitivity ≥ 95%, (2) specificity ≥ 95%, and (3) number of parameters ≤ 5M.

**Figure 3:**
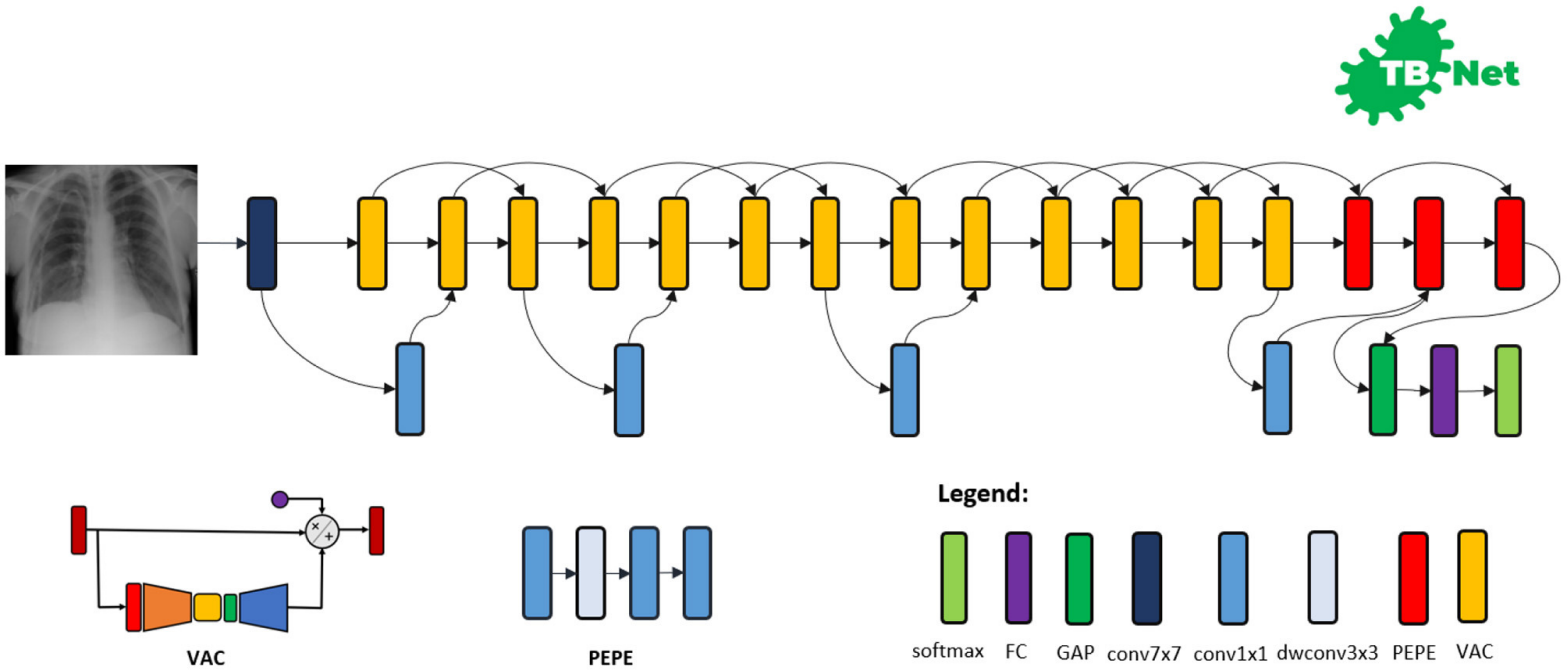
The proposed TB-Net architecture design. The TB-Net design exhibits high architectural heterogeneity, light-weight design patterns, and the utilization of visual attention condensers, with macro-architecture and micro-architecture designs tailored specifically for the detection of TB cases from chest X-ray images.

The proposed TB-Net self-attention deep convolutional neural network design is shown in Figure 3. A number of interesting observations can be made. First, it can be observed that the overall network architecture design exhibits high macro-architecture and micro-architecture heterogeneity, with a mix of standard convolutions, depth-wise convolutions, point-wise convolutions, and self-attention mechanisms with different micro-architecture characteristics. This high degree of architectural diversity and heterogeneity reflects the fact that a machine-driven design exploration strategy was leveraged to customize the design in a very fine-grained manner specifically around TB case detection using CXR images to achieve an optimal level of performance for the given task at hand.

Second, it can be observed that the network architecture possesses a very light-weight design, consisting primarily of highly efficient depth-wise convolutions and point-wise convolutions as well as leveraging light-weight design patterns such as project-expansion-projection-expansion (PEPE) patterns that reduces and expands representational dimensionality in a way that strikes an optimal balance between accuracy and efficiency. This light-weight design pattern utilization reflects the ability for a machine-driven design exploration strategy to tailor the macro-architecture design of deep neural network architectures based on the architectural complexity constraint imposed in the indicator function. The highly efficient architecture design of TB-Net is especially critical to enabling potential widespread adoption since real-world TB screening scenarios in high-risk regions faced by poverty and economic distress have high resource and cost constraints, and so deployment would often have to take place on low-cost, low-end computing devices.

Third, it can be observed that a majority of the layers of the TB-Net network architecture design is comprised of visual attention condensers (Wong et al., 2020b), which are a variant of the highly efficient attention condenser self-attention mechanisms recently introduced in Wong et al. (2020a). More specifically, visual attention condensers produce condensed embedding characterizing joint spatial and cross-channel activation relationships and achieves selective attention accordingly to improve representational capability while maintaining very low architectural and computational complexity. As a result, by leveraging visual attention condensers, the proposed TB-Net network architecture design facilitates for high TB screening performance by better focusing its attention on the distinguishing visual cues within CXR images for identifying TB positive patient cases in a very efficient manner.

Last but not least, the end-stage sub-architecture of the TB-Net deep neural network architecture consists of a global average pooling layer, a fully-connected layer, and a softmax layer to produce the final output for predicting whether a patient is TB positive or negative. The TB-Net network is available in an open source manner at https://github.com/darwinai/TuberculosisNet.

### 3.3. Network Training

Training was conducted on the proposed TB-Net deep neural network architecture design using stochastic gradient descent optimization with a learning rate of 0.0001, momentum of 0.9, and a batch size of 8 for 200 epochs.

Each image went through the following pre-processing steps. To begin, images were cropped such that 5% of the top, left, and right sides of the image were removed, and 25% of the bottom was removed. Each image was then resized back to a size of 224 ×224 pixels. Based on our experiments, a size of 224 ×224 pixels was able to provide optimal performance and retained sufficient textural information to discriminate between TB positive and TB negative patients, with no performance gains for higher resolutions. This is consistent with Sabottke and Spieler (2020), noting that the performance of neural networks plateau after a certain resolution as sufficient information is available for high accuracy.

Next, data augmentation was conducted on training images with the following augmentation types: horizontal flip, random cropping (within 10%), random contrast shift (within 20%), and random intensity shift (within 10%). Test and validation images were not augmented. These augmentations were chosen as they are typical transformations in medical image applications, as CNNs are good at learning spatial filters that capture spatially local discriminant features. Since spatially local visual biomarkers and patterns within the lungs that are useful for characterizing tuberculosis may have similar visual appearance across patients but are not identical and have geometric and intensity variations, the use of geometric transformations can allow a neural network to better generalize.

Following the augmentation step, for all images, a mask was applied on the top-left and top-right corners to remove pre-existing markers on the images, and filled with black. Finally, the images were normalized to the range 0–1, and the previously masked corners were filled in with the average pixel value across the entire dataset. These pre-processing steps were designed such that any noise, pre-existing markings, and unnecessary areas of the image were removed prior to model training.

All construction, training, and evaluation are conducted in the TensorFlow deep learning framework. The scripts for the aforementioned process are available in an open source manner at https://github.com/darwinai/TuberculosisNet.

### 3.4. Explainability-Driven Performance Validation

To gain a deeper insight and validate the decision-making behavior of TB-Net, we leveraged GSInquire (Lin et al., 2019), a state-of-the-art explainability method that was shown to no only provide explanations that better reflect the decision-making behavior of deep neural networks when compared to other well-known methods, but also identify specific critical factors that are quantitatively critical to the decision-making process rather than relative heatmaps pertaining to relative importance variations.

Briefly, GSInquire leverages an inquisitor ℐ within a generator-inquisitor pair {G,ℐ} during the generative synthesis (Wong et al., 2018) process used in the machine-driven exploration strategy. The inquisitor can be more formally defined as $ℐ(G;\theta_{ℐ})$ parameterized by $\theta_{ℐ}$ that, given a generator G, produces $\Delta \theta_{G}$ (i.e., $\Delta \theta_{G}=ℐ(G)$). To obtain an interpretation *z* of a decision from a network N=G(∫) comprising a set *V* of vertices *v*∈*V* and a set *E* of edges *e*∈*E* (here, the TB-Net network) for an input signal *x* (here, a CXR image), the inquisitor ℐ probes $\left\{V_{s},{ℰ}_{s}\right\}$, where $V_{s}\subseteq V_{s}$ and ${ℰ}_{s}\subseteq E_{s}$ with targeted stimulus *x*, and the resulting set $Y_{G\left(s\right)}$ of reactionary responses $y\in Y_{G\left(s\right)}$ are observed and used to update *I*. After the update of *I*, $\Delta \theta_{G}=ℐ(G)$ is generated, transformed, and projected into same subspace as *x via* a transformation $T(\Delta \theta_{G\left(s\right)})$ to create an interpretation *z*(*x*; *N*). The details related to the use of GSInquire to generate interpretations of deep neural network decision-making behavior for CXR images can be found in Wang et al. (2020). Here, the interpretation *z* indicates the critical factors leveraged by TB-Net in its decision-making process for a CXR image.

Explainability-driven performance validation facilitates for: (1) transparent validation of the TB-Net network to ensure that the TB case detection process is primarily driven by clinical relevant visual indicators such as infiltrates, consolidations, pleural effusion, cavities, and lesions, (2) identification of potentially erroneous visual indicators being leveraged such as embedded markers and text, imaging artifacts, and motion artifacts, and (3) improve greater trust in the clinical workflow through greater transparency.

### 3.5. Radiologist Validation

The results for TB-Net that were obtained during the explainability-driven performance validation process for select patient cases are further reviewed and reported on by two board-certified radiologists (AS and AA). The first radiologist (AS) has over 10 years of experience, and the second radiologist (AA) has over 19 years of radiology experience.

## 4. Results

We evaluate the efficacy of the proposed TB-Net self attention deep convolutional neural network design for detecting TB cases from CXR images in three ways. First, we evaluate the quantitative performance of the network as well as study its architectural and computational complexity. Second, we study its decision-making behavior using an explainability-driven performance validation strategy. Third, we conduct radiologist validation on study the consistency of TB-Net's decision-making behavior with radiologist interpretation. The details of the quantitative and qualitative results are shown below. Further exploration of the results will be discussed in Section 5, along with an explanation of how TB-Net is able to achieve higher performance without the need for high architectural and computational complexity.

### 4.1. Quantitative Analysis

**Figure 4:**
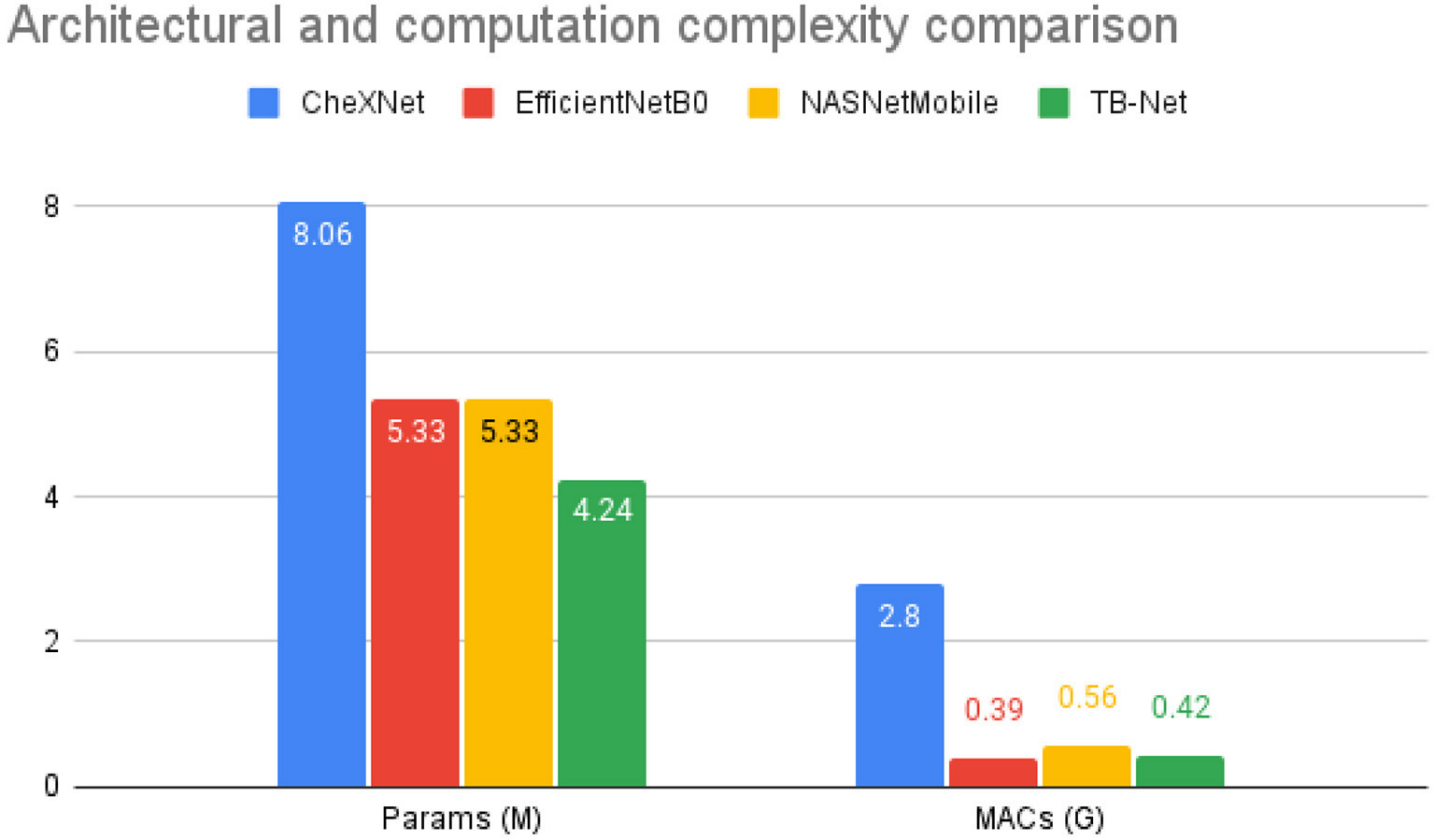
Architectural and computational complexity comparison between the CheXNet (Rajpurkar et al., 2017) architecture vs. the proposed TB-Net architecture. As shown, TB-Net achieves ~1.9 × fewer parameters and ~6.7 × lower MACs.

The accuracy, sensitivity, and specificity of the proposed TB-Net are shown in Table 1, while the architectural and computational complexity of the proposed TB-Net are shown in Figure 4. For comparison purposes, CheXNet (Rajpurkar et al., 2017) was also evaluated, given that it is a state-of-the-art deep neural network architecture for CXR image analysis and was found in a comprehensive study conducted by Rahman et al. (2020) to be the best performing deep neural network architecture amongst nine different deep convolutional neural network architectures [ResNet-18 (He et al., 2016), ResNet-50, ResNet-101, ChexNet (Rajpurkar et al., 2017), Inception-V3 (Szegedy et al., 2015), VGG-19 (Simonyan and Zisserman, 2015), DenseNet-201 (Huang et al., 2018), SqueezeNet (Iandola et al., 2016), and MobileNet-v2 (Sandler et al., 2019)] for the task of detecting TB patient cases from CXR images without the use of segmentations. In addition, the EfficientNetB0 (Tan and Le, 2019) and NASNetMobile (Zoph et al., 2018) model architectures were also evaluated, chosen due to the fact that these models were also created using state-of-the-art neural architecture search for optimal performance.

**Table 1:** Accuracy, sensitivity, and specificity of TB-Net on the test data from the multi-national patient cohort.

**Architecture**	**Accuracy**	**Sensitivity**	**Specificity**
	**(%)**	**(%)**	**(%)**
CheXNet (Rajpurkar et al., 2017)	99.42	**100**	98.85
EfficientNetB0 (Tan and Le, 2019)	98.99	99.42	98.56
NASNetMobile (Zoph et al., 2018)	99.28	98.84	**99.71**
TB-Net	**99.86**	**100**	**99.70**

*Better performance metric in **bold***.

The following three equations below outline how the accuracy, sensitivity, and specificity values were calculated for each model, respectively. A *true positive* (TP) is a TB positive patient correctly classified as TB positive, a *false negative* (FN) is a TB positive patient incorrectly classified as TB negative, a *false positive* (FP) is a TB negative patient incorrectly classified as TB positive, and finally a *true negative* (TN) is a TB negative patient correctly classified as TB negative.


(2) $$Accuracy=\frac{TP+TN}{TP+TN+FP+FN}$$

(3) $$Sensitivity=\frac{TP}{TP+FN}$$

(4) $$Specificity=\frac{TN}{FP+TN}$$

### 4.2. Qualitative Analysis

**Figure 5:**
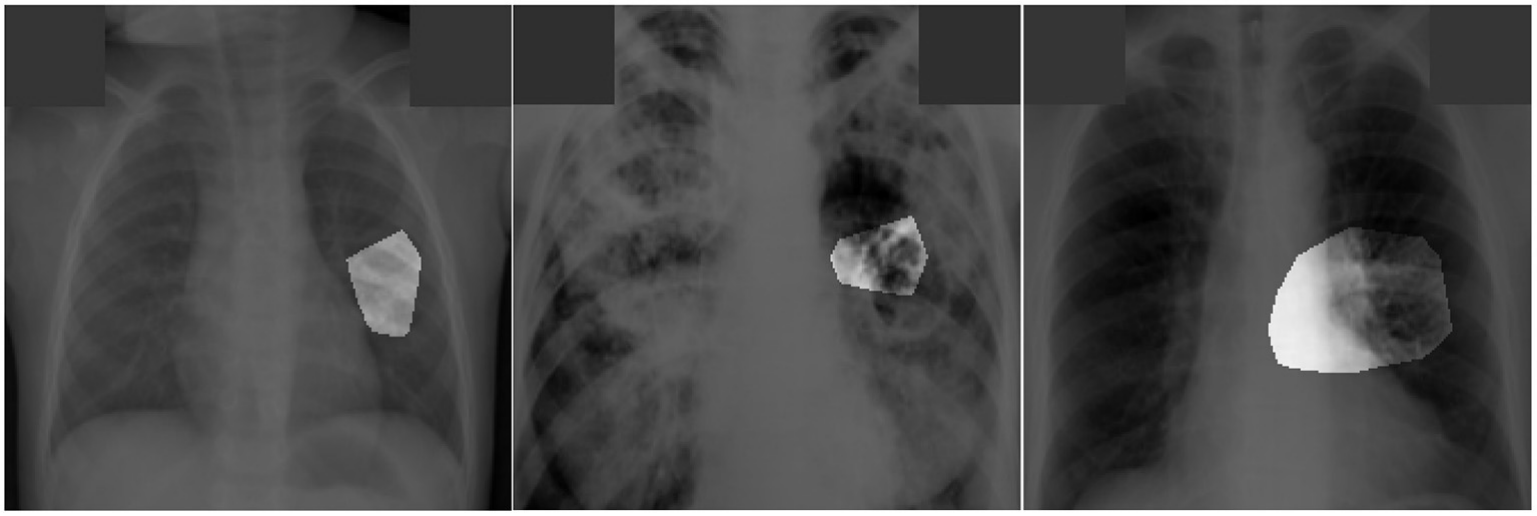
Examples of patient cases with associated critical factors (highlighted regions) as identified by GSInquire (Lin et al., 2019) during explainability-driven performance validation. From **left** to **right**: (a) Case 1, (b) Case 2, and (c) Case 3. Radiologist validation showed that several of the critical factors identified are consistent with radiologist interpretation.

Explainability-driven performance validation was conducted on TB-Net and examples of patient cases with associated critical factors identified by GSInquire for driving the decision-making behavior of the proposed TB-Net are shown in Figure 5. It can be seen that the proposed TB-Net is primarily relying on clinically relevant areas of the lung in the CXR images to drive its decision-making behavior.

### 4.3. Radiologist Analysis

The expert radiologist findings for select patient cases in regards to the relevancy of the critical factors identified during the explainability-driven performance validation process as shown in Figure 5 are as follows. In all three cases, TB-Net correctly detected them to be TB positive cases.

**Case 1**. According to radiologist findings, both radiologists did not observe any abnormalities that would be indicative of TB, while identified critical factors leveraged by TB-Net indicate some form of abnormality in the right midlung region.

**Case 2**. According to radiologist findings, it was observed by both radiologists that there is a cavity in the hilar region that coincide with the identified critical factors leveraged by TB-Net in that region. One of the radiologists also observed other scattered opacities in the lungs.

**Case 3**. According to radiologist findings, both radiologists did not observe any abnormalities that would be indicative of TB, while identified critical factors leveraged by TB-Net indicate some form of abnormality in the right lower lung region.

## 5. Discussion

From the quantitative results shown in Table 1, a number of observations can be made. First, it can be observed from Table 1 that the TB-Net network achieved high accuracy, sensitivity, and specificity of 99.86, 100, and 99.7%, respectively, and thus achieves the same level of sensitivity and slightly higher accuracy and specificity when compared to CheXNet used in this study. When compared to EfficientNetB0, TB-Net also achieves higher accuracy, sensitivity, and specificity across all three metrics. Only NASNetMobile has a similar specificity as TB-Net, but does so at the cost of lower accuracy and sensitivity. The high sensitivity achieved with the proposed TB-Net implies that there would be fewer missed TB positive patients during the TB screening process, which is highly desirable from a clinical perspective especially given the infectious nature of TB and the need to reduce spread within the community. On the other hand, the high specificity achieved with the proposed TB-Net implies that there would be fewer false positive detections, which is important to reduce the burden on healthcare systems caused by additional work for clinicians and front-line healthcare workers.

Second, it can be observed from Figure 4 that the TB-Net network achieves low architectural complexity and computational complexity of 4.24 million parameters and 0.42 billion multiply-accumulate (MAC) operations, which is also ~1.9 × lower and ~6.7 × lower, respectively, than that of CheXNet used in this study. The high architectural and computational efficiency achieved by the proposed TB-Net network is important for enabling CAD-driven TB screening on low-cost, low-power computing devices, particularly given the types of resource-constrained clinical environments faced in high-risk regions faced by poverty and economic distress. For comparison, other highly efficient network architectures such as EfficientNet or NasNetMobile consist of 5.3 million parameters, demonstrating the lightweight aspect of TB-Net, which only contains 4.24 million. TB-Net also only requires a similar number of MAC operations as these state-of-the-art model architectures, further showcasing the ability of machine-driven architecture design in generating powerful yet compact networks.

These quantitative results illustrate that the self-attention network architecture design tailored *via* a machine-driven design exploration approach is capable of achieving high detection performance while maintaining high efficiency, thus illustrating high potential for resource-constrained clinical environments. The addition of visual attention condensers is what enables this high performance while maintaining a low complexity, as they allow TB-Net to much more efficiently focus attention on the most important visual bio-markers for characterizing tuberculosis phenotypes, and more effectively learn the difference between tuberculosis positive and negative patients.

Quantitative results can also be seem from Figure 5. In this image, it can be seen that the proposed TB-Net is primarily relying on clinically relevant areas of the lung in the CXR images to drive its decision-making behavior. Furthermore, it can be seen that it is not relying on erroneous visual indicators such as motion artifacts, embedded symbols and text, and imaging artifacts. As such, it can be seen that TB-Net is exhibiting clinically relevant decision-making behavior.

The third part of our evaluation procedure involved validation from certified radiologists. Based on the radiologist findings and observations on the three patient cases, it was shown that the critical factors identified by GSInquire as critical factors driving the decision-making behavior of TB-Net was consistent with radiologist interpretation for Case 2, but not all regions of concern as identified by the radiologists are necessarily leveraged by TB-Net in making its TB case detection decisions. Furthermore, more interestingly, the critical factors identified by GSInquire as critical factors driving the decision-making behavior of TB-Net for Case 1 and Case 3 for correctly determining the patients as TB positive were not identified by the radiologists, which could lead to more interesting insights and methods for tuberculosis detection in the future.

## 6. Conclusion

In this study, we introduced TB-Net, a self-attention deep convolutional neural network tailored for tuberculosis case screening. A machine-driven design exploration strategy was leveraged to build a highly customized deep neural network architecture with attention condensers. An explainability-driven performance validation process was conducted to validate TB-Net's decision-making behavior, and was further confirmed *via* radiologist validation. Experimental results demonstrate that TB-Net can not only achieve high tuberculosis case detection performance in terms of sensitivity and specificity, but also exhibit clinically relevant behavior during an explainability-driven performance validation process as well as during the radiologist validation process for the case where radiologists identified anomalies.

Since tuberculosis is an on-going global health crisis and is curable if detected, the hope is that research such as TB-Net and open source initiatives such as the COVID-Net initiative that TB-Net is part of can accelerate the advancement and adoption of deep learning-driven computer aided diagnosis solutions within a clinical setting to aid front-line health workers and healthcare systems in improving clinical workflow efficiency and effectiveness in the fight against the on-going tuberculosis crisis in high-risk regions where there is a tremendous scarcity of experienced human readers for tuberculosis screening. Therefore, additional care was taken to perform explainability-driven performance validation as well as radiologist validation to conduct additional checks and balance around the decision-making behavior of TB-Net in a transparent and responsible manner.

To the best of the authors' knowledge, this research on tuberculosis screening using deep learning does not put anyone at any potential disadvantages. However, it is important to note that TB-Net is not a production-ready solution and the current focus is on facilitating research advancements in the area. While not a production-ready solution, the hope is that the open-source release of TB-Net as part of the COVID-Net initiative will support researchers, clinicians, and citizen data scientists in advancing this field in the fight against this global public health crisis. Further work involves the exploration of tailored deep neural network designs for other tasks in the tuberculosis clinical workflow (e.g., severity assessment and treatment planning), as well as exploring a system that can differentiate between tuberculosis and SARS-CoV-2 infections as they share similar symptoms.

## Data Availability Statement

The original contributions presented in the study are included in the article/supplementary material, further inquiries can be directed to the corresponding author/s.

## Author Contributions

AW conceived the experiments. JL, HR-K, and HL conducted the experiments. AA and AS reviewed and reported on select patient cases and corresponding explainability results illustrating model's decision-making behavior. All authors analyzed the results and reviewed the manuscript and contributed to the article and approved the submitted version.

## Conflict of Interest

AW, JL, HR-K, and HL are affiliated with DarwinAI Corp. The remaining authors declare that the research was conducted in the absence of any commercial or financial relationships that could be construed as a potential conflict of interest.

## Publisher's Note

All claims expressed in this article are solely those of the authors and do not necessarily represent those of their affiliated organizations, or those of the publisher, the editors and the reviewers. Any product that may be evaluated in this article, or claim that may be made by its manufacturer, is not guaranteed or endorsed by the publisher.
